# Bacterial Profile and Antibiotic Susceptibility Patterns of Chronic Otitis Media at a Tertiary Care Center in Maharashtra

**DOI:** 10.7759/cureus.67989

**Published:** 2024-08-28

**Authors:** Savya Vaid, Rashmi P Rajashekhar, Heer Shah, Manu Babu, Aditi S Moruskar

**Affiliations:** 1 Otolaryngology - Head and Neck Surgery, Dr. D. Y. Patil Medical College, Hospital & Research Centre, Dr. D. Y. Patil Vidyapeeth (Deemed to be University), Pune, IND; 2 Microbiology, Dr. D. Y. Patil Medical College, Hospital & Research Centre, Dr. D. Y. Patil Vidyapeeth (Deemed to be University), Pune, IND

**Keywords:** antibiotic susceptibility, culture, bacteria, hearing loss, chronic otitis media

## Abstract

Background

Chronic otitis media (COM) is a persistent inflammatory condition of the middle ear and mastoid cavity, characterized by otorrhea and a perforated tympanic membrane. The microbial agents associated with COM vary over time and by geographic location. According to the WHO classification of countries based on COM prevalence, India is classified as having the highest prevalence (>4%) due to a national average of 7.8%, with the majority of the burden affecting individuals in lower socioeconomic strata.

Objectives

The objective of this study is to analyze the bacterial profile and antibiotic susceptibility patterns of isolates in patients with COM and to examine the age and gender distribution of these patients.

Methods

A cross-sectional microbiological study was conducted in the Department of Otorhinolaryngology at Dr. D. Y. Patil Medical College, Hospital & Research Centre, Dr. D. Y. Patil Vidyapeeth, Pune (Deemed to be University), Pune, India, from January 2023 to March 2023. The sample size was determined using WinPepi software. A total of 30 samples were collected from patients aged 5-15 years with active COM who presented to the ENT OPD. Patients who had been treated with antibiotics in the 15 days prior to the study were excluded.

Results

Of the 30 patients, most were in the age groups of 16-25 years (36.67%) and 26-35 years (16.67%), with only 10% in the 5-15 years age group. The age range of participants was eight to 55 years, with a mean age of 27 years. There was a gender disparity in the distribution of COM, with males representing 56.67% and females 43.33%, resulting in a male-to-female ratio of 1.3:1. The majority of patients had mucosal COM (24/30, 80%), while the remaining had squamosal COM (6/30, 20%). *Pseudomonas aeruginosa* was isolated in 24 samples (80%), and *Staphylococcus aureus* was identified in six samples (20%).

Conclusion

In the Indian context, where many predisposing factors for COM are prevalent, the disease remains highly common and can lead to severe complications if not treated promptly. This study analyzed ear swabs from 30 patients diagnosed with COM. The results revealed that *P. aeruginosa* was the most common microorganism, followed by *S. aureus*. The most effective antibiotics against *P. aeruginosa* were piperacillin-tazobactam, ceftazidime, and meropenem. For *S. aureus*, the most effective antibiotics included teicoplanin, vancomycin, linezolid, and cotrimoxazole.

## Introduction

Chronic otitis media (COM) is a persistent inflammatory disease of the middle ear and mastoid cavity, characterized by otorrhea and a perforated tympanic membrane [1]. The etiology of COM involves a variety of microbial agents, with significant variation depending on time and geographical location. Recent years have seen a prevalence of aerobic bacteria such as *Pseudomonas aeruginosa *and *Staphylococcus aureus*. The microbiology of COM can vary greatly due to factors such as geographical location, individual immunity, and antibiotic use [2].

Several factors contribute to the development of COM, including repeated respiratory infections, poor living standards and hygiene, lack of access to proper medical care, irrational use of antibiotics, malnutrition, allergies, and overcrowding [3]. According to the WHO, India has the highest prevalence of COM (>4%), with a national average of 7.8%, primarily affecting the lower socioeconomic strata [4,5]. Globally, the incidence of COM is 4.76%, equating to approximately 31 million cases. COM is a major cause of preventable hearing impairment, particularly in resource-limited settings [6]. In the pediatric population, impaired hearing can lead to delays in language development and poor school performance. Untreated COM can result in severe complications such as mastoiditis, facial nerve palsy, labyrinthitis, meningitis, and brain abscess [7].

While the introduction of antibiotics has significantly reduced these complications compared to the pre-antibiotic era, the irrational use of antibiotics has led to the emergence of drug-resistant strains, making COM increasingly difficult to treat and leading to a resurgence of its complications. Therefore, our objective is to study the bacterial profile and antibiotic susceptibility patterns of isolates in patients with COM to enable appropriate treatment and prevent complications.

## Materials and methods

A cross-sectional microbiological study was conducted in the Department of Otorhinolaryngology at Dr. D. Y. Patil Medical College, Hospital & Research Centre, Dr. D. Y. Patil Vidyapeeth, Pune (Deemed to be University), Pune, India. Thirty samples were collected from patients with active COM attending the outpatient department. Inclusion criteria were patients aged five to 55 years diagnosed with either mucosal or squamosal COM. Exclusion criteria included patients who had been treated with antibiotics in the past 15 days or those diagnosed with otitis externa or otomycosis.

After obtaining informed verbal consent, a thorough history was recorded, including basic demographic data and clinical details. An otoscopic examination was performed, and a single sample was collected from the discharging ear using a sterile cotton swab under aseptic conditions. The swab was immediately transported to the microbiology lab for processing.

In the lab, bacterial identification was carried out using Gram staining, culturing on blood and MacConkey agars, and biochemical testing. The bacterial cultures were then subjected to antibiotic susceptibility testing using the Kirby-Bauer disc diffusion method, with results interpreted according to Clinical & Laboratory Standards Institute guidelines [8]. Data were compiled and analyzed using percentages, frequencies, and Chi-squared tests with IBM SPSS Statistics for Windows, Version 21.0 (Released 2012; IBM Corp., Armonk, NY, USA), with statistical significance set at p < 0.05.

## Results

Once the 30 suitable patients were selected for the study, ear swabs were collected and sent for culture and antibiotic sensitivity testing. The patients met the inclusion criteria for COM. After statistical analysis, the results were tabulated by organism isolated (Table 1), age and sex distribution (Table 2), affected ear, and type of COM (Table 3). The antibiograms of the isolated organisms were compiled based on the number of resistant and sensitive strains found (Table 4, Table 5).

**Table 1:** Distribution of bacterial isolates The bacteriological analysis identified two types of causative organisms. *P. aeruginosa *was isolated in 24 samples (80%), while* S. aureus *was found in six samples (20%). This result is highly significant, with p < 0.0001.

Organism isolated	Frequency (n = 30)	Percentage
Pseudomonas aeruginosa	24	80%
Staphylococcus aureus	6	20%

**Table 2:** Age and sex distribution of COM The majority of patients were in the age groups of 16-25 years (36.66%) and 26-35 years (16.67%), while only 10% were in the 5-15 years age group. The age range of patients was 8-55 years, with a mean age of 27 years. Males constituted 56.67% of the patients, while females made up 43.33%. Despite the observed differences in age and sex distribution, these findings are not statistically significant, with p-values of 0.0801 for age group and 0.4386 for sex distribution. COM, chronic otitis media

Age group (years)	Frequency (n = 30)	Percentage
5-15	3	10%
16-25	11	36.66%
26-35	5	16.67%
36-45	4	13.34%
46-55	7	23.33%
Male	17	56.67%
Female	13	43.33%

**Table 3:** Ear affected and type of COM COM was most commonly observed in the left ear (50%), followed by the right ear (46.67%), with only one patient having bilateral involvement (p = 0.0001, highly significant). The predominant type of COM was mucosal (80%), while the remaining cases were of the squamosal type (20%) (p < 0.0001, highly significant). COM, chronic otitis media

Ear involved	Frequency (n = 30)	Percentage
Left	15	50%
Right	14	46.67%
Bilateral	1	3.34%
Type of COM	Frequency (n = 30)	Percentage
Mucosal	24	80%
Squamosal	6	20%

**Table 4:** Antibiotic sensitivity pattern of Pseudomonas aeruginosa For *P. aeruginosa*, a highly significant difference (p < 0.0001) was observed in antibiotic sensitivity. Ceftazidime and piperacillin-tazobactam were highly effective, with over 90% of the isolates showing sensitivity. Cefepime, cefoperazone, and tobramycin were also effective but had a lower sensitivity rate, affecting only 40-50% of isolates. Ciprofloxacin, a commonly used antibiotic, showed sensitivity in 50% of isolates but had overall unsatisfactory results, with 48.2% resistance and 1.2% intermediate sensitivity. Even last-resort drugs like amikacin and meropenem revealed some resistance pockets, raising concerns about their effectiveness.

S. no.	Antibiotic	Number of sensitive strains	Resistant strains	Percentage of sensitive strains
1	Ceftazidime	23	1	95.80%
2	Ciprofloxacin	12	11	50%
3	Cefepime	18	3	75%
4	Cefoperazone	18	2	75%
5	Piperacillin-tazobactem	23	1	95.80%
6	Tobramycin	18	6	75%
7	Amikacin	17	7	70.80%
8	Meropenem	23	1	95.80%
9	Colistin	24	0	100%

**Table 5:** Antibiotic sensitivity pattern of Staphylococcus aureus For *S. aureus*, a significant p-value of 0.0005 was noted for its antibiotic sensitivity pattern. MSSA was isolated in three samples, while MRSA was found in the remaining samples. Cotrimoxazole demonstrated superior sensitivity, with over 83% of isolates showing effectiveness. Higher-class antibiotics such as teicoplanin, vancomycin, and linezolid also yielded good results. In contrast, penicillin G, clindamycin, and gentamicin showed lower sensitivity, ranging from 45-50%. Ciprofloxacin was ineffective, with none of the strains showing sensitivity. MRSA, methicillin-resistant* Staphylococcus aureus*; MSSA, methicillin-sensitive *Staphylococcus aureus*

S. no.	Antibiotic	Number of sensitive strains	Resistant strains	Percentage of sensitive strains
1	Ciprofloxacin	0	6	0%
2	Cotrimoxazole	5	1	83.34%
3	Erythromycin	1	5	16.67%
4	Penicillin G	3	3	50%
5	Clindamycin	3	3	50%
6	Gentamicin	3	1	50%
7	Teicoplanin	6	0	100%
8	Vancomycin	6	0	100%
9	Linezolid	6	0	100%

## Discussion

COM is a significant cause of preventable hearing loss and is influenced by multiple factors. It is notably more prevalent in rural areas of India, as observed in various studies [9]. In this study, 30 patients with active COM were examined, with over 50% being in the second and third decades of life, the mean age being 27 years, and 56.67% being male. This is consistent with findings from Mishra, who reported that over 60% of cases were in the 21-30 years age group [10]. This higher prevalence in younger age groups might be attributed to increased exposure or delays in seeking treatment. Similar results were observed by Narve et al., who found that 57% of COM patients were male [11].

In this study, 80% of the cases were of the mucosal (safe) type of COM, while the remaining were squamosal (unsafe). This is aligned with Ramya et al., who found 88.2% of their 188 patients had mucosal COM and 11.2% had squamosal COM [12]. The predominant organisms isolated were* P. aeruginosa *(80%) and *S. aureus*. This finding is consistent with a study by Nazir and Kadri, which identified *P. aeruginosa *and *S. aureus *as the primary pathogens, followed by other aerobic bacteria such as *Proteus*, *Escherichia coli*, and *Klebsiella *in Kashmir. Another study of 94 patients also found *P. aeruginosa *and *S. aureus *to be the most common isolates, with a few anaerobic gram-positive bacteria [13].

Antimicrobial susceptibility testing showed that most* P. aeruginosa *strains were sensitive to ceftazidime and piperacillin-tazobactam (87.5%), followed by cefepime and tobramycin. However, ciprofloxacin showed high resistance, with 50% of strains unresponsive. A 2017 study by Juyal et al. also highlighted that piperacillin-tazobactam and amikacin were most effective against *P. aeruginosa*, while ciprofloxacin, tobramycin, and gentamicin showed high resistance [14]. Similar findings were reported, with most strains being sensitive to piperacillin-tazobactam, meropenem, amikacin, and cefoperazone [15].

For *S. aureus*, cotrimoxazole, teicoplanin, and vancomycin showed good results, while penicillin G and gentamicin had mixed outcomes, with 50% sensitivity. Ciprofloxacin and erythromycin showed high resistance, with over 90% of isolates being resistant. This is consistent with other studies where over 90% of *S. aureus* strains were sensitive to cotrimoxazole and gentamicin [16] and more than 65% of strains were resistant to ciprofloxacin [17].

In India, ciprofloxacin and amoxicillin are commonly prescribed for discharging ears. Recent studies indicate a shift toward higher-class antibiotics due to the indiscriminate use of common antibiotics, overtreatment, improper dosage, self-medication, and lack of routine culture sensitivity tests.

This study identified *P. aeruginosa *as the most common organism in COM patients. Immediate initiation of antibiotic sensitivity testing is crucial to prevent complications associated with COM. The study’s limitation is the small sample size.

## Conclusions

Almost all the predisposing factors for COM are prevalent in the Indian context, making it a highly common disease that can lead to severe complications if not treated promptly. This study analyzed ear swabs from 30 patients diagnosed with COM. The results indicated that *P. aeruginosa *is the most common pathogen, followed by *S. aureus*. The most effective antibiotics against *P. aeruginosa *were piperacillin-tazobactam, ceftazidime, and meropenem, while teicoplanin, vancomycin, linezolid, and cotrimoxazole were most effective against *S. aureus*. Both pathogens showed significant resistance to ciprofloxacin, a commonly used antibiotic. This study underscores the importance of routine antibiotic sensitivity testing for chronic ear infections and regular antibiotic surveillance in hospitals to ensure appropriate antibiotic use and to manage the spread of resistant strains.

## References

[1] Browning GG, Weir J, Kelly G, Swan IR (2018). Chronic otitis media. Scott-Brown’s Otorhinolaryngology and Head and Neck Surgery.

[2] Gupta P, Varshney S, Kumar SK, Mohanty A, Jha MK (2020). Chronic suppurative otitis media: a microbiological review of 20 years. Indian J Otol.

[3] Qureshi S, Rehman U (2015). Demographic influences on complicated chronic suppurative otitis media. Indian J Otol.

[4] World Health Organization (2004). Chronic suppurative otitis media: burden of illness and management options. Chronic suppurative otitis media Burden of illness and management Options.

[5] Parvez A, Khan Z, Hashmi SF (2016). A cross sectional study of chronic suppurative otitis media and its associated factors among primary school children in rural and urban areas of Aligarh, India. Int J Community Med Public Health.

[6] Sangeetha S, Shiva Kumar S, Vijayakarthikeyan M (2020). A cross sectional study on chronic suppurative otitis media (CSOM) among patients attending a tertiary care centre in Salem, Tamil Nadu. Natl J Res Community Med.

[7] Khairkar M, Deshmukh P, Maity H, Deotale V (2023). Chronic suppurative otitis media: a comprehensive review of epidemiology, pathogenesis, microbiology, and complications. Cureus.

[8] Weinstein MP, Lewis JS 2nd (2020). The clinical and laboratory standards institute subcommittee on antimicrobial susceptibility testing: background, organization, functions, and processes. J Clin Microbiol.

[9] Sandip MP, Sood A, Hamjol SC (2018). Prevalence of chronic suppurative otitis media in school going children. Indian J Otol.

[10] Mishra S (2020). A study on chronic suppurative otitis media in a tribal area medical college. Int J Curr Microbiol Appl Sci.

[11] Narve VP, Gupta P, Basu R, Sachan M, Jhawar K, Freni JK, Punjabi M (2013). Clinicopathological study of 100 cases of chronic suppurative otitis media in tertiary health care centre. J Evol Med Dent Sci.

[12] Ramya SP, Subramani P, Sukumaran R (2020). A cross sectional study on the bacteriologic pattern of chronic otitis media and its antibiotic sensitivity. Int J Otorhinolaryngol Head Neck Surg.

[13] Nazir A, Kadri S (2014). Aerobic bacteriology of chronic suppurative otitis media: a hospital based study. Int J Res Med Sci.

[14] Juyal D, Sharma M, Negi V (2017). Pseudomonas aeruginosa and its sensitivity spectrum in chronic suppurative otitis media: a study from Garhwal Hills of Uttarakhand state, India. Indian J Otol.

[15] Thakur P, Poorey V (2015). Clinicomicrobiological evaluation and antibiotic susceptibility in cases of chronic suppurative otitis media. Indian J Otol.

[16] Shamim R, Gopi A (2018). A study of bacterial and fungal isolates of chronic suppurative otitis media with their antibiotic susceptibility pattern in patients attending a tertiary care hospital. J Pure Appl Microbiol.

[17] Soumya TR, Ravi D (2017). Bacteriological profile of chronic suppurative otitis media in a tertiary care hospital. Int J Otorhinolaryngol Head Neck Surg.

